# Dielectric Barrier Discharge Ionization in Characterization of Organic Compounds Separated on Thin-Layer Chromatography Plates

**DOI:** 10.1371/journal.pone.0106088

**Published:** 2014-08-29

**Authors:** Michał Cegłowski, Marek Smoluch, Michał Babij, Teodor Gotszalk, Jerzy Silberring, Grzegorz Schroeder

**Affiliations:** 1 Department of Supramolecular Chemistry, Faculty of Chemistry, Adam Mickiewicz University in Poznan, Poznań, Poland; 2 Department of Biochemistry and Neurobiology, Faculty of Materials Science and Ceramics, AGH-University of Science and Technology, Krakow, Poland; 3 Faculty of Microsystem Electronics and Photonics, Wroclaw University of Technology, Wroclaw, Poland; 4 Center for Polymer and Carbon Materials, Polish Academy of Sciences, Zabrze, Poland; University of Edinburgh, United Kingdom

## Abstract

A new method for on-spot detection and characterization of organic compounds resolved on thin layer chromatography (TLC) plates has been proposed. This method combines TLC with dielectric barrier discharge ionization (DBDI), which produces stable low-temperature plasma. At first, the compounds were separated on TLC plates and then their mass spectra were directly obtained with no additional sample preparation. To obtain good quality spectra the center of a particular TLC spot was heated from the bottom to increase volatility of the compound. MS/MS analyses were also performed to additionally characterize all analytes. The detection limit of proposed method was estimated to be 100 ng/spot of compound.

## Introduction

Thin-layer chromatography (TLC) is a very simple, cost-effective and fast chromatographic technique allowing separation of most chemical mixtures [1]. Detection of separated compounds relies mostly on their optical visualization using UV light or appropriate reagents, such as Dragendorff reagent. The information obtained by these means is, however very limited. For definite identification of the compound, which has been visualized, it must be compared with a standard that has been simultaneously eluted on TLC. Coupling TLC and mass spectrometry (MS), which is a very useful tool for structural analysis of organic compounds, would make a simple and easy to operate technique. That is why the application of mass spectrometry in characterization of compounds separated on TLC plates has been a subject of interest for many scientists [2], [3] and recently some reviews on this topic have been published [4], [5], [6], [7]. The TLC-MS methods can be divided into indirect sampling TLC-MS and direct sampling TLC-MS [5].

Indirect sampling TLC-MS is of less importance because it requires time-consuming processes, such as scratching of the spot containing particular compound, followed by solvent extraction of the adsorbed substance [8]. The direct sampling TLC-MS techniques allow for mass spectrometric analysis of compounds directly from the surface of TLC plates. These methods can be further divided into vacuum-based and ambient TLC-MS approaches [5].

Desorption/ionization methods that operate under vacuum have been used to obtain mass spectra of compounds directly from the surface of TLC plates. Several techniques such as fast atom bombardment (FAB) [9], [10], secondary ion mass spectrometry (SIMS) [11], laser desorption (LDI) [12], matrix-assisted laser desorption/ionization (MALDI) [13], [14], surface-assisted laser desorption/ionization (SALDI) [15] have been applied to characterize compounds separated by TLC. These techniques however, generate several problems: obtaining high vacuum in the ion source considerably increases the time of analysis, volatile compounds have poor sensitivity, MALDI matrices produce interferences with mass spectra, poor reproducibility in quantitative analysis, diffusion of analytes on TLC plates after applying MALDI matrix solution [16].

Some of the presented problems were solved by coupling ambient MS techniques with TLC. Hence, the TLC plates do not need to be placed in a vacuum chamber for ionization, and the analysis is much faster without the risk that volatile compounds will desorb from the plate before MS analysis. Moreover, the TLC plate size is not limited by the dimensions of a vacuum chamber. Techniques, such as electrospray ionization (ESI) [17], [18], [19], electrospray-assisted laser desorption ionization (ELDI) [20], desorption electrospray ionization (DESI) [21], laser desorption/atmospheric pressure chemical ionization (LD-APCI) [22], atmospheric pressure matrix-assisted laser desorption/ionization (AP-MALDI) [23], [24] and direct analysis in real time (DART) [25], [26] have been adopted to generate mass spectra of compounds directly from TLC plates.

Dielectric barrier discharge ionization (DBDI) produces low-temperature plasma by dielectric barrier discharges (DBD) [27]. DBD are obtained at ambient conditions and are formed between two electrodes with a dielectric layer that separates them. The presence of a dielectric layer limits the average current in the gas space, which causes formation of the low-temperature plasma containing a large number of high energy electrons [28], [29]. DBD plasma was used to produce mass spectra of compounds desorbed from different surfaces [30], to detect nonvolatile chemicals directly on various surfaces [31], utilized as an ion source for liquid chromatography/mass spectrometry [32], and coupled *on line* with TLC for mercury speciation [33].

Herein we report the coupling of DBD plasma source with TLC. DBDI is capable of providing very fast and selective ionization of each particular compound separated on TLC plate. The ionized compounds can be characterized using MS and MS/MS modes. Combination of these approaches can find many applications, particularly in synthetic organic laboratories and forensic sciences.

## Materials and Methods

### Synthesis

**Figure 1:**
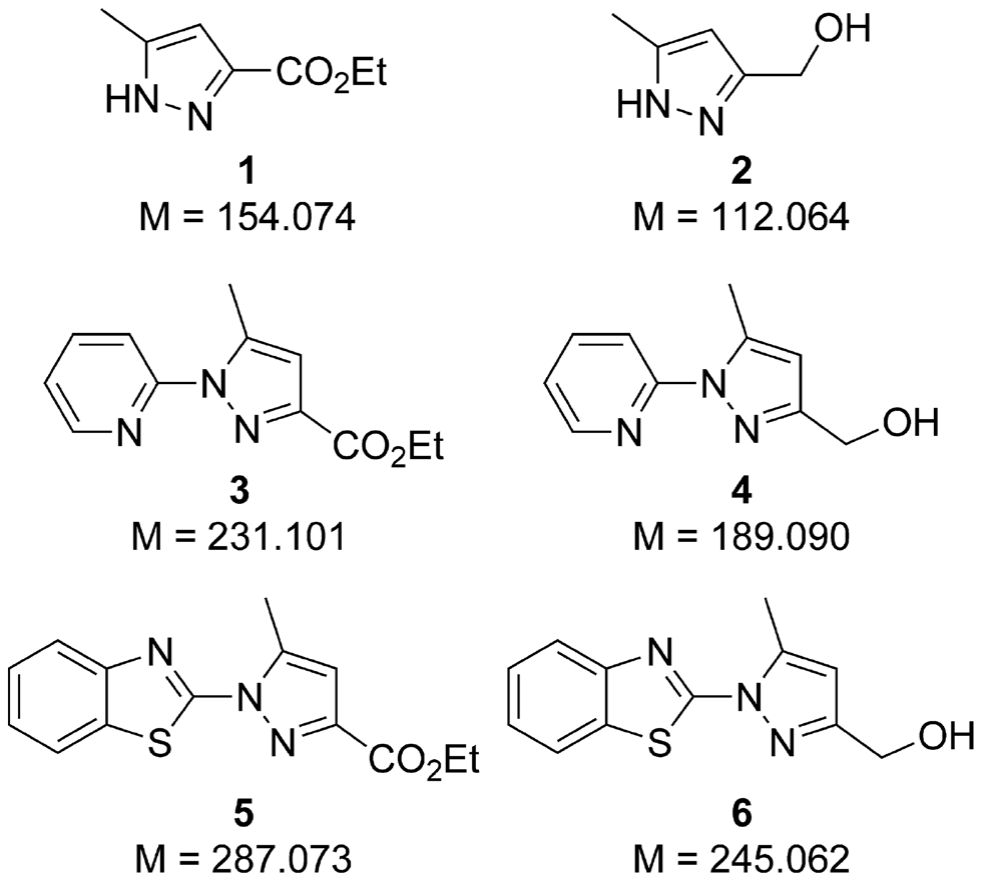
Structures of compounds separated on TLC plates.

Synthesis of compounds **1–6**, whose structures are presented in Figure 1, as well as information about reagents used are given in the **Information S1**.

### Instrumental Design

The DBD plasma source consists of quartz capillaries (O.D. 1.5 mm and I.D. 0.8 mm). The electrodes of 2 mm width were made of copper rings surrounding the capillary tube and the gap between the inner edges was 5 mm. The grounded electrode was placed 6 mm apart from the capillary end. The plasma was operated with helium (99.996% purity) flow of 1 L/min, by applying a voltage of 8 kV. Additional data describing DBD ion source are given in details elsewhere [34]. A Bruker Esquire 3000 quadrupole ion trap mass spectrometer (Bruker Daltonics, Bremen, Germany) was used for all measurements. The typical ESI-MS source settings were found to be optimal also for the DBDI source, with the exception of the mass spectrometer entrance glass capillary voltage, where lower potential (1 kV) compared to the standard ESI setting (4.5 kV) was used. The temperature of the glass capillary was set to 200°C, the drying gas flow was maintained at 3 L/min., and the nebulizer gas (N_2_) was not applied. The scan range was set from 80 to 500 m/z. For MS/MS experiments the isolation width was set to 2 m/z and the fragmentation amplitude was in the range of 0.5 to 0.8 unit.

### TLC preparation and detection

**Figure 2:**
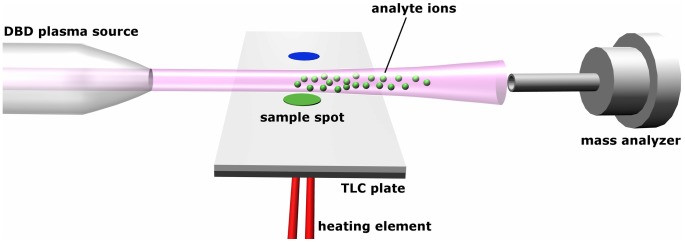
Schematic illustration of DBDI coupled to TLC.

**Figure 3:**
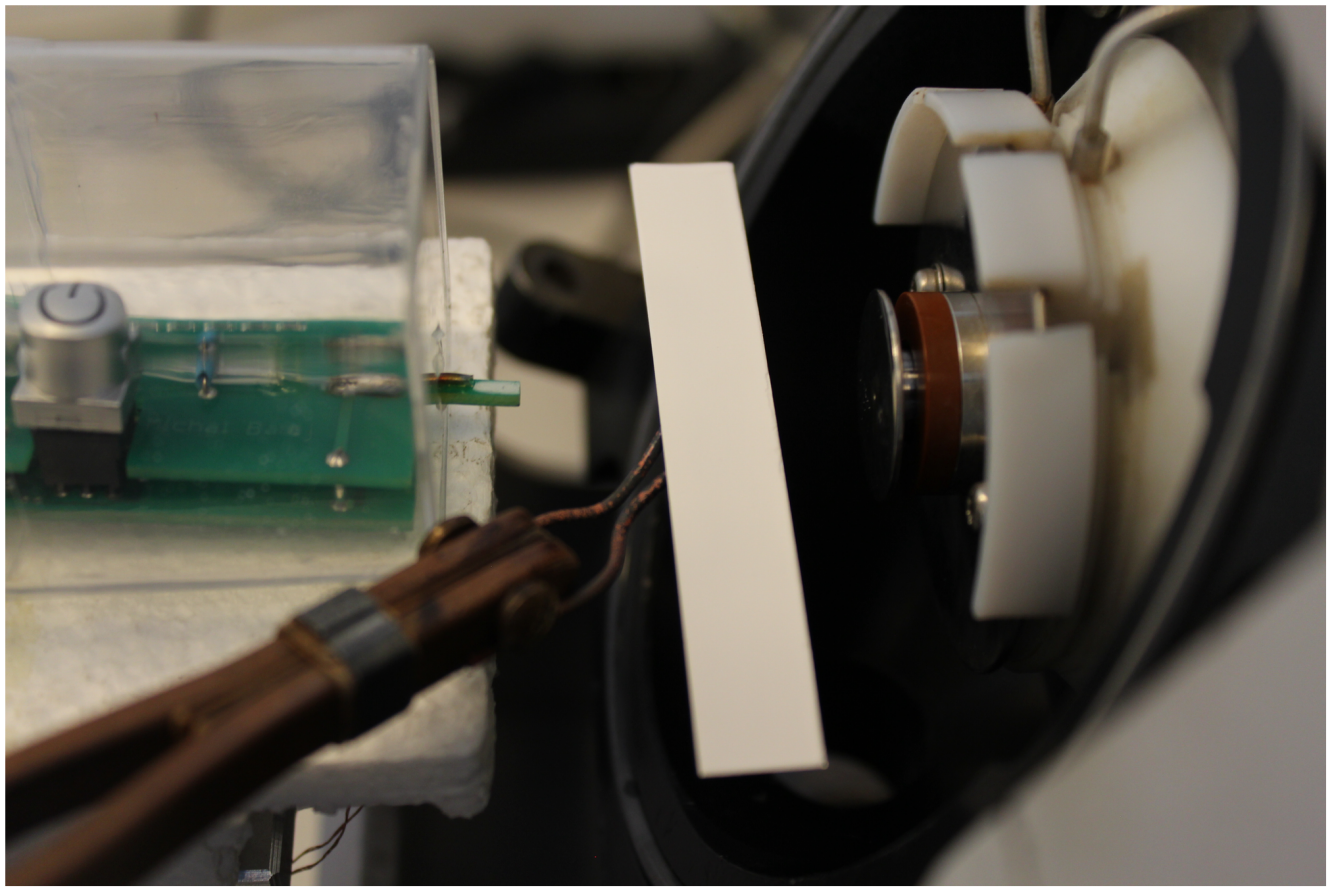
Photograph of experimental setup used for TLC-MS analysis.

Compounds **1–6** were dissolved in dichloromethane to obtain final concentration of 10 mg/mL. One µL sample solutions were spotted on a TLC plate (Merck Millipore TLC silica gel 60 F_254_ aluminium sheets). The compounds were spotted in ester-alcohol pairs on a single TLC plate. The plates with compounds **1** and **2** were developed with CH_2_Cl_2_/Et_2_O (1∶1, v/v) solution, those with **3** and **4** were developed with CH_2_Cl_2_/Et_2_O (5∶1, v/v), those with **5** and **6** were developed with CH_2_Cl_2_/Et_2_O (10∶1, v/v). The plate containing all six compounds was developed with CH_2_Cl_2_/Et_2_O (10∶1, v/v). For MS analysis, the spots were visualized on TLC with UV light and marked with a pencil. The TLC plate was then cut through the centers of all spots, resulting as a narrow strip, and a heating element, of a diameter similar to the size of a spot, was attached to the bottom of a particular spot. The strip was then heated from the bottom and held manually so that the plasma released from DBD ion source would emerge just above the marked spot. The heating element consisted of resistance wire which temperature was constantly increased in the range 25°C–400°C using manual control until the signal of particular analyte was visible on mass spectra. Each analysis has been completed in less than one minute. After each m/z measurement the heating element was allowed to cool down. It was then attached to the bottom of the next spot and the measurements were continued. Figure 2 displays a schematic illustration of coupling DBDI to TLC, whereas Figure 3 shows a photograph of experimental setup used for TLC-MS analysis.

## Results and Discussion

**Figure 4:**
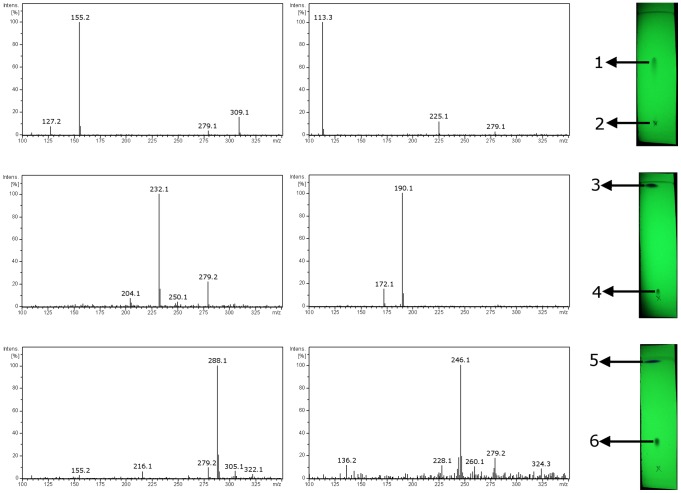
Photographs of TLC plates (visualized in UV light) with corresponding mass spectra obtained from particular spots. The spots were assigned to the label of particular compound. Left panel represents the mass spectra of compounds with higher *R_f_* value (esters).

To examine the performance of DBDI in the on-spot detection of compounds separated on TLC plates, solutions of six compounds were deposited onto plates. The compounds were applied in ester-alcohol pairs on a single TLC plate, because these pairs are resolved properly on TLC and alcohols are products of ester reduction. The spectra obtained from three TLC plates with the six compounds are presented in Figure 4. Each spectrum represents the major ion corresponding to the protonated molecule with little or no background ions. The most intense signals belonging to contaminants are observed at *m/z* 279.1 and 205.1 and derive from di-*n*-butyl phthalate (DBP) [35], which is commonly used as a plasticizer in plastics, from which DBD ion source has been manufactured. Particularly, the tube used to transport helium from gas cylinder to DBD ion source, is made of polyvinyl chloride (PVC) for which DBP is used as a plasticizer. We therefore, believe that the possible source of contamination is desorption of DBP by helium stream. An advantage of DBDI technique is its ability to produce intact (usually protonated) species with little or no fragmentation what substantially simplifies identification of the compounds separated on TLC.

To show that good quality mass spectra can be generated even when compounds are poorly resolved on TLC, all six compounds were deposited and separated on a single TLC plate. The resulting mass spectra of all six compounds are presented in the **Information S1**. Each spectrum obtained represents the major ion corresponding to the protonated molecule of a particular compound with only small traces of other compounds resolved on TLC.

**Figure 5:**
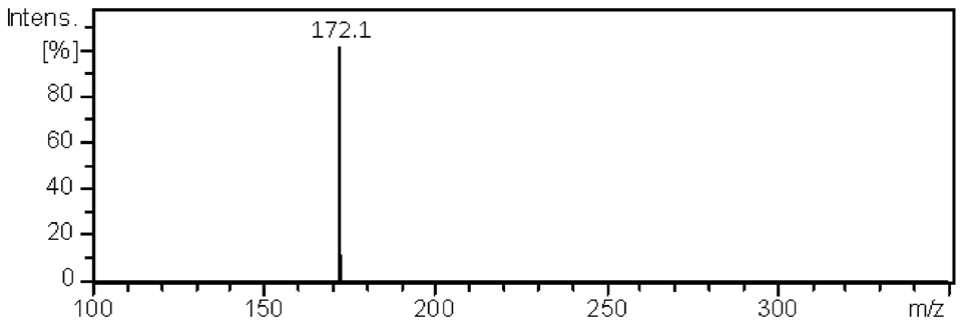
MS/MS spectrum of the signal at *m/z* 190.1 derived from compound 4.

The structures of the analytes were confirmed by MS/MS analysis of all compounds. The exemplary MS/MS spectrum of the signal at *m/z* 190.1 (assigned to protonated compound **4**) is presented in Figure 5. Only one daughter ion ([**4**+H – H_2_O]^+^  =  *m/z* 172.1) appeared in the mass spectrum. MS/MS spectra of all compounds examined are presented in the **Information S1**.

**Figure 6:**
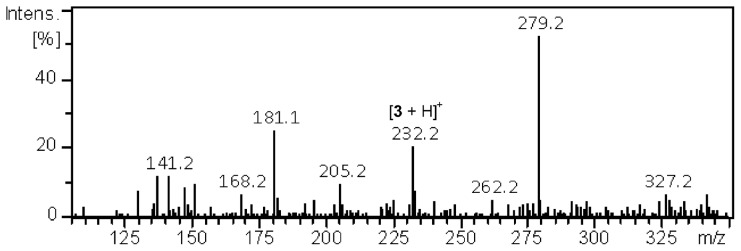
Mass spectrum of compound 3 (100 ng/spot) recorded directly from TLC spot.

The detection limits of compounds deposited on TLC plates analyzed by DBD ion source were estimated using the solutions containing different concentrations of compounds **1–6**. Each solution was deposited onto TLC plate and after air-drying the plate was heated as described in the Methods section and introduced into the gas stream. The best result was obtained for compound **3** where 100 ng/spot was detected. However, in the spectrum obtained (presented in Figure 6) the most intense signal comes from DBP contamination. Nevertheless, this result is sufficient for TLC-MS analysis because the amount of 100 ng/spot of compound **3** could not be seen under the UV light, therefore this amount is far less than needed for common TLC analysis. It is worth noting, that this is only semi-quantitative analysis because the ion intensity of an analyte desorbed from TLC plate varies depending on the thickness and composition of silica gel, temperature, diffusion coefficient of the analyte on the TLC plate during mass analysis and positioning of TLC plate in the gas stream.

## Conclusions

In this paper we demonstrated the use of DBDI in conjunction with TLC separations. This technique appeared to be effective for direct analysis of compounds that can be separated by thin layer chromatography. The coupling of DBDI and TLC has many advantages, such as ionization under ambient conditions, low-cost of a DBD ion source, easy and fast analysis of volatile and semi-volatile compounds, unlimited dimensions of TLC plate and fast sample switching. These advantages make DBDI a robust and convenient mass spectrometric technique, which allows for fast screening of TLC plates that are run every day in many laboratories.

## Supporting Information

Figure S1 **Photograph of TLC plate (visualized under UV light), on which all six compounds have been separated, combined with mass spectra obtained from respective spots.** The spots and the mass spectra were assigned to the number labeling of particular compound.
(TIF)

Figure S2 **MS/MS spectra of ions at: a)** ***m/*** **z 155.2 (assigned to protonated compound 1); b)** ***m/*** **z 113.3 (assigned to protonated compound 2); c)** ***m/*** **z 232.1 (assigned to protonated compound 3); d)** ***m/*** **z 288.1 (assigned to protonated compound 5); e)** ***m/*** **z 246.1 (assigned to protonated compound 6).**
(TIF)

Information S1 **Information about reagents used and synthesis of compounds 1–6.**
(DOCX)
