# Supplementary figures and images for: Dielectric Barrier Discharge Ionization in Characterization of Organic Compounds Separated on Thin-Layer Chromatography Plates
Source: PLoS One. 2014 Aug 29;9(8):e106088. doi: 10.1371/journal.pone.0106088 (PMC4149504; doi:10.1371/journal.pone.0106088)

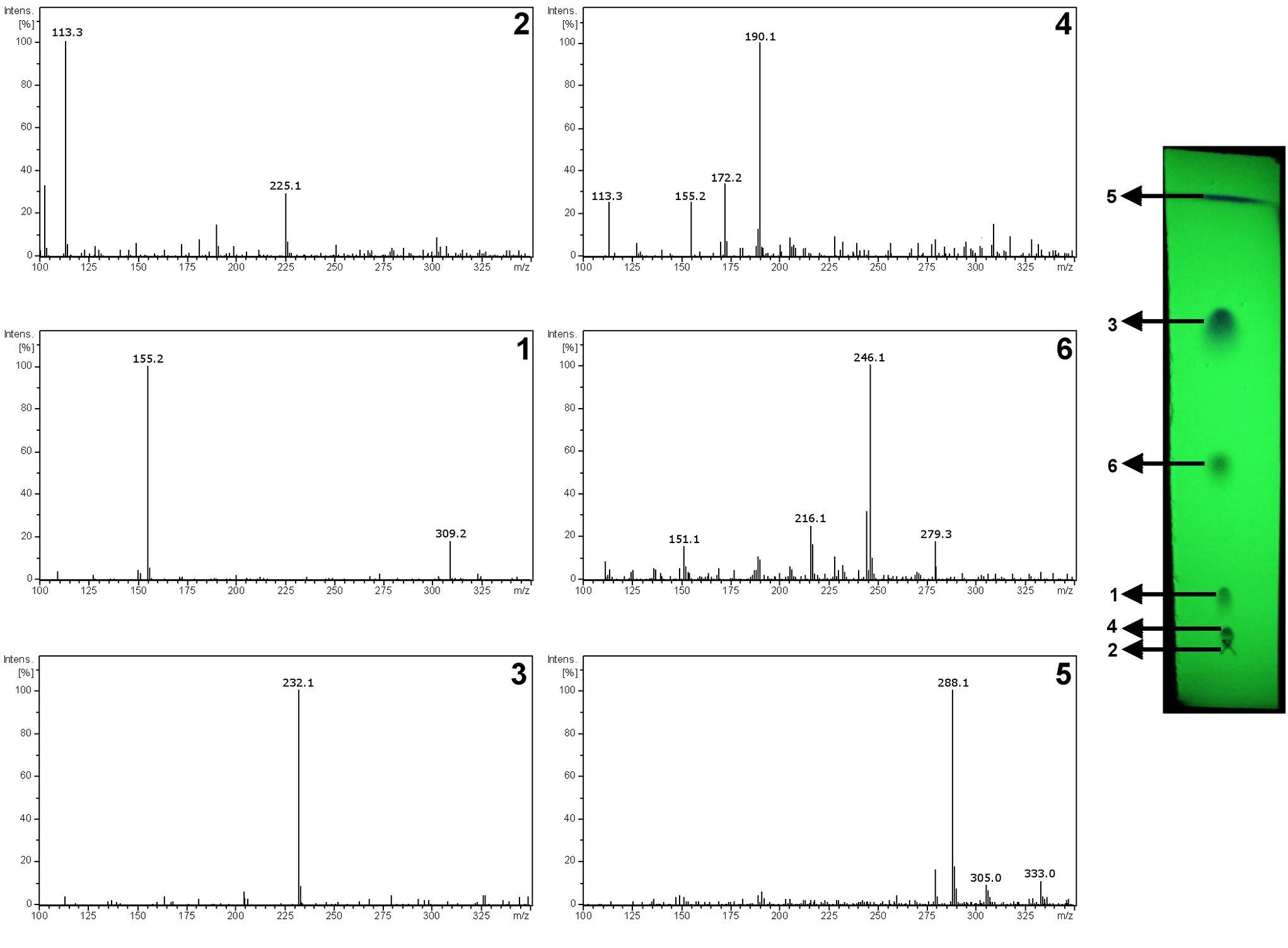

Supplement: Figure S1 — Photograph of TLC plate (visualized under UV light), on which all six compounds have been separated, combined with mass spectra obtained from respective spots. The spots and the mass spectra were assigned to the number labeling of particular compound. (TIF) [file pone.0106088.s001.tif]

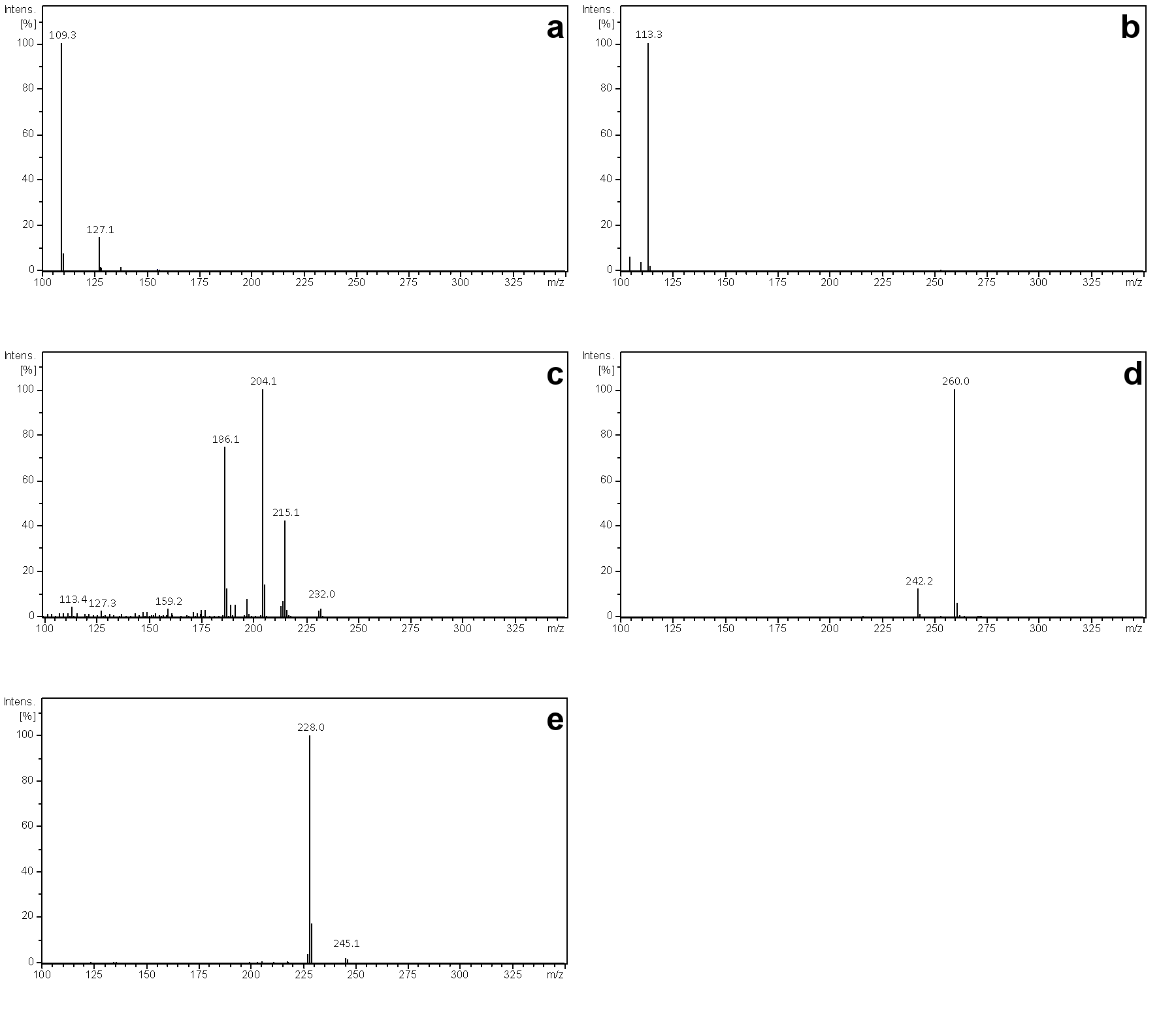

Supplement: Figure S2 — MS/MS spectra of ions at: a) m/ z 155.2 (assigned to protonated compound 1); b) m/ z 113.3 (assigned to protonated compound 2); c) m/ z 232.1 (assigned to protonated compound 3); d) m/ z 288.1 (assigned to protonated compound 5); e) m/ z 246.1 (assigned to protonated compound 6). (TIF) [file pone.0106088.s002.tif]
