# Supplementary material for: Dielectric Barrier Discharge Ionization in Characterization of Organic Compounds Separated on Thin-Layer Chromatography Plates
Source: PLoS One. 2014 Aug 29;9(8):e106088. doi: 10.1371/journal.pone.0106088 (PMC4149504; doi:10.1371/journal.pone.0106088)
Supplement: Information S1 — Information about reagents used and synthesis of compounds 1–6. (DOCX) [file pone.0106088.s003.docx]

**Information S1. Information about reagents used and synthesis of compounds 1 – 6.**

**Reagents**

All reagents used were obtained from commercial sources. Ethyl 2,4-dioxovalerate, hydrazine hydrate, 2-hydrazinopyridine, 2-hydrazinobenzothiazole, lithium aluminum hydride, anhydrous tetrahydrofuran (THF) were obtained from Sigma-Aldrich (St. Louis, MO, USA). Anhydrous ethyl alcohol and all other solvents were of the purity grade p.a., obtained from POCH (Gliwice, Poland) and were used without further purification.

**Synthesis of compound 1**

To the solution of ethyl 2,4-dioxovalerate (2 g, 12.6 mmol) in absolute ethanol (30 mL), a mixture of 40% hydrazine solution in water (1.6 g, 12.8 mmol) and few drops of concentrated hydrochloric acid in absolute ethanol (20 mL) were added dropwise. The mixture was than heated at reflux for 5 h. The solvent was removed under reduced pressure and few milliliters of distilled water were added to the residue. The residue was neutralized with solution of sodium carbonate. The solution was extracted three times with dichloromethane. The extracts were combined and dried with anhydrous sodium sulfate. Solvent was eliminated under reduced pressure yielding compound **1**.

^1^H NMR (CDCl_3_) δ: 1.37 (t, 3H, -CH_2_-C**H_3_**); 2.37 (s, 3H, -C**H_3_**); 4.37 (q, 2H, -C**H_2_**-CH_3_); 6.59 (s, 1H, Pz-**H)**; 7.71 (bs, 1H, N_Pz_-**H**).

**Synthesis of compound 2**

To a suspension of LiAlH_4_ (0.1 g, 2.6 mmol) in anhydrous THF (20 mL) cooled to 0°C, a solution of compound **1** (0.19 g, 1.2 mmol) in anhydrous THF (30 mL) was added dropwise. After the entire solution of compound **1** has been added, the mixture was stirred for additional 1 h at 0°C. The mixture was then refluxed for 2 h. The excess of LiAlH_4_ was neutralized by slow addition of water (0.1 mL) 15% aqueous solution of sodium hydroxide (0.2 mL) and water (0.3 mL). The resulting mixture was stirred for 3 h. The solid material was filtered off and washed with hot THF. The filtrate and THF washings were combined and the solvent was removed under reduced pressure yielding compound **2**.

^1^H NMR (CDCl_3_) δ: 2.29 (s, 3H, -C**H_3_**); 4.48 (bs, 2H, -O**H**, N_Pz_-**H**); 4.66 (s, 2H, -C**H_2_**-OH); 6.00 (s, 1H, Pz-**H**).

**Synthesis of compound 3**

Compound **3** was synthesized according to the previously described method.^1^ To a solution of ethyl 2,4-dioxovalerate (5 g, 31.6 mmol) in absolute ethanol (35 mL), a mixture of 2-hydrazinopyridine (3.45 g, 31.6 mmol) and concentrated hydrochloric acid (1 g) in absolute ethanol (70 mL) were added dropwise. The mixture was than heated to 60°C for 5 h. The solvent was removed under reduced pressure and few milliliters of distilled water were added to the residue. The residue was neutralized by a solution of sodium carbonate. The resulting solution was extracted three times with dichloromethane. The extracts were combined and dried with anhydrous sodium sulfate. Solvent was eliminated under reduced pressure and the crude product was purified by column chromatography on a silica gel with dichloromethane/diethyl ether (10:1) as the eluent.

^1^H NMR (CDCl_3_) δ: 1.41 (t, 3H, CH_2_-C**H_3_**); 2.68 (s, 3H, -C**H_3_**); 4.42 (q, 2H, -C**H_2_**-CH_3_); 6.71 (s, 1H, Pz-**H**); 7.27 (m, 1H, Py-**H**β); 7.85 (m, 1H, Py-**H**γ); 7.97 (m, 1H, Py-**H**δ); 8.47 (m, 1H, Py-**H**α).

**Synthesis of compound 4**

Compound **4** was synthesized according to literature [1]. To the suspension of LiAlH_4_ (0.62 g, 16.3 mmol) in anhydrous THF (20 mL) cooled to 0°C, a solution of compound **3** (1.88 g, 8.1 mmol) in anhydrous THF (30 mL) was added dropwise. After the entire solution of compound **3** has been added, the mixture was stirred for additional 30 minutes at 0°C. The excess of LiAlH_4_ was neutralized by the slow addition of water (0.65 mL) 15% aqueous solution of sodium hydroxide (1.3 mL) and water (1.95 mL). The resulting mixture was stirred for 3 h. The solid material was filtered off and washed with hot THF. The filtrate and THF washings were combined and the solvent was removed under reduced pressure yielding compound **4**.

^1^H NMR (CDCl_3_) δ: 2.65 (s, 3H, -C**H_3_**); 4.71 (s, 2H, -C**H_2_**-OH); 6.19 (s, 1H, Pz-**H**); 7.19 (m, 1H, Py-**H**β); 7.81 (m, 2H, Py-**H**γ, δ); 8.44 (m, 1H, Py-**H**α).

**Synthesis of compound 5**

A solution of ethyl 2,4-dioxovalerate (2.87 g, 18.2 mmol), 2-hydrazinobenzothiazole (3 g, 18.2 mmol) and concentrated hydrochloric acid (0.75 g) in absolute ethanol (120 mL) was refluxed for 5 h. The solvent was removed under reduced pressure and few milliliters of distilled water were added to the residue. The residue was neutralized by a solution of sodium carbonate. The resulting solution was extracted three times with dichloromethane. The extracts were combined and dried with anhydrous sodium sulfate. Solvent was eliminated under reduced pressure and the crude product was purified by column chromatography on a silica gel with dichloromethane/diethyl ether (100:1) as the eluent.

^1^H NMR (CDCl_3_) δ: 1.43 (t, 3H, -CH_2_-C**H_3_**); 2.86 (s, 3H, -C**H_3_**); 4.43 (q, 2H,-C**H_2_**-CH_3_); 6.74 (s, 1H, Pz-**H**); 7.39 (t, 1H, Ph-**H**); 7.49 (t, 1H, Ph-**H**); 7.86 (d, 1H, Ph-**H**); 7.93 (d, 1H, Ph-**H**).

**Synthesis of compound 6**

To a suspension of LiAlH_4_ (0.17 g, 4.5 mmol) in anhydrous THF (15 mL) cooled to 0°C, a solution of compound **3** (0.61 g, 2.1 mmol) in anhydrous THF (15 mL) was added dropwise. After the entire solution of compound **3** has been added, the mixture was stirred for additional 30 minutes at 0°C. The excess of LiAlH_4_ was neutralized by the slow addition of water (0.2 mL) 15% aqueous solution of sodium hydroxide (0.4 mL) and water (0.6 mL). The resulting mixture was stirred for 3 h. The solid material was filtered off and washed with hot THF. The filtrate and THF washings were combined and the solvent was removed under reduced pressure yielding compound **6**.

^1^H NMR (CDCl_3_) δ: 1.87 (bs, 1H, -O**H**); 2.82 (s, 3H, -C**H_3_**); 4.73 (s, 2H, -C**H_2_**-OH); 6.26 (s, 1H, Pz-**H**); 7.34 (t, 1H, Ph-**H**); 7.45 (t, 1H, Ph-**H**); 7.82 (d, 1H, Ph-**H**); 7.89 (d, 1H, Ph-**H**).

REFERENCES

1. Radi S, Attayibat A, Ramdani A, Lekchiri Y, Hacht B, et al. (2007) C,N‐Pyridylpyrazole‐Based Ligands: Synthesis and Preliminary Use in Metal Ion Extraction. Sep Sci Technol 42: 3493-3501.
